# Using immunohistochemistry to classify the molecular subtypes of Paget's disease of the breast

**DOI:** 10.1002/cam4.6066

**Published:** 2023-05-11

**Authors:** Yujiao Cai, Zhongquan Cheng, Jiarui Nangong, Xiaodan Zheng, Zhu Yuan

**Affiliations:** ^1^ Department of General Surgery Capital Medical University, Beijing Friendship Hospital Beijing China; ^2^ Department of Pathology Capital Medical University, Beijing Friendship Hospital Beijing China

**Keywords:** breast cancer, immunohistochemistry, molecular subtype, Paget's disease of the breast

## Abstract

**Purpose:**

To classify the molecular subtypes of Paget's disease of the breast, and then compare them with general breast cancer to get deeper understanding of this disease and offer better management of associate patients in clinical decisions.

**Methods:**

We used immunohistochemistry to examine 42 cases of this disease by antibodies against estrogen and progesterone receptors, Ki‐67, as well as human epidermal growth factor receptor 2 (HER‐2). Due to damage and loss of specimens, etc., we obtained 36 pathological specimens from the 42 patients. For 30 of 36 pathological specimens (83.3%), we obtained a complete molecular subtype. Cause the other 6 pathological specimens have missing immunohistochemistry items. For patients with bilateral breast cancer, only information on the side with PDB is listed. For patients with recurrence, only information on the first onset was included. We finally compared and studied the molecular subtype of 26 samples. We calculated the relative frequencies of molecular subtypes including luminal A, luminal B, HER‐2‐enriched, and basal‐like and compared them between PDB and general breast carcinomas in other studies.

**Results:**

The luminal A and B subtype were found, respectively, in 3 (11.5%) and 6 (23.1%) of all patients, and 15 cases of HER‐2‐enriched subtype was detected (57.7%). In addition, 2 (7.7%) showed a basal‐like subtype.

**Conclusion:**

The molecular subtypes of common breast cancer and PDB‐associated breast cancer differ. Luminal subtypes are the most common in the former, while within our samples HER‐2 positive subtype was the highest in PDB‐associated breast carcinoma. With further understanding of this disease, rational therapies will be applied in different patients and cures for PDB and PDB‐associated carcinoma will be achieved.

## Abstract 2

We examined molecular subtype distribution in Paget's disease of the breast (PDB). Subtypes HER‐2, luminal A, and luminal B were seen in 57.7%, 11.5%, and 23.1% cases. Triple‐negative subtype (basal‐like) was found in 7.7% cases. Molecular subtype of associated breast carcinoma was similar to that of PDB. Molecular subtypes vary between PDB‐associated and general breast carcinoma.

## 1 INTRODUCTION

Paget's disease of the breast (PDB) is a rare skin malignancy first described in 1874 by James Paget as “an eczematous nipple changes before latent invasive breast cancer”.^1^ Among all primary breast cancer, PDB only epidemiologically accounts for 0.7%–4.3%^2^ and is histologically characterized by a Paget cells (PCs) infiltration with abundant pale cytoplasm and nuclei located centrally in the epidermal layer of the nipple‐areolar complex (NAC).^3^ The clinical presentation of PDB is usually eczematous changes or ulceration of NAC, with scaling, bleeding, crusting, or oozing often with pain or pruritus in most patients.^4^, ^5^ PDB can be divided into the following categories: (1) PDB with invasive ductal carcinoma (IDC); (2) PDB with underlying ductal carcinoma in situ (DCIS); (3) PDB alone without IDC or DCIS.^6^ According to previous analyses of (SEER) data conducted between 1973 and 1987, more than half of PDB patients had concurrent underlying IDC.^7^, ^8^

There are two theories about pathogenesis of PDB: the epidermotropic and the transformation theories.^9^ The Epidermotropic theory postulates that ductal cancer cells that migrate along the basal membrane of the nipple are the origin of Paget's cells.^10^ In contrast, the latter suggests that Paget cells originate from malignant transformation of keratinocytes, which would give rise to PDB.^9^, ^10^, ^11^, ^12^ Currently surgery is performed as the basic therapy for PDB in clinical settings. Mastectomy with sentinel lymph node biopsy or axillary lymph node dissection has been the standard of care for decades.^9^ Breast‐conserving surgery is a good option for patients with no palpable mass and benign mammogram or low microvessel density (MVD).^10^, ^13^, ^14^, ^15^ In addition, the use of chemotherapy and hormone therapy as adjuvant treatment is based on tumor subtypes and tumor staging.^16^, ^17^ In short, PDB prognosis ultimately depends on its molecular subtype, commonly HER‐2+ which has poorer prognosis than luminal subtype.^10^

Immunohistochemistry is a powerful tool in detecting the molecular subtypes of PDB contributing to the staging, therapy selection and prognosis prediction of this disease, meanwhile in differentiated from other nipple‐associated lesions including nipple adenoma, malignant melanoma, pagetoid Spitz nevus, florid papillomatosis of the nipple, and so on.^18^, ^19^, ^20^, ^21^ In our study we use a immunohistochemical profile including ER, PR, Ki‐67, and HER‐2 to exam thoroughly the molecular subtypes of PDB which can deep our understanding of this rare disease. Based on classifying different subtypes of PDB, rational therapies will be applicated in clinical cases and a better cure for the disease can be achieved.

## 2 MATERIALS AND METHODS

### 2.1 Patients information

Through the pathology retrieval system of Beijing Friendship Hospital Affiliated to Capital Medical University, we analyzed PDB cases diagnosed and archived from 2011 to 2020.We collected 42 PDB patients from more than 2000 breast cancer patients. We obtained relevant information of the patients by consulting the medical record system and follow‐up, including the patient's gender, age, tumor location in the left or right breast, lesion size, lymph node metastasis, etc. PDB diagnosis was validated by two pathologists (Xiaodan Zheng and Shuhong Zhang) specialized in breast pathology. Tissues were collected from surgically excised specimens from patients, embedded in paraffin, sectioned using a microtome, and stained using hematoxylin and eosin.

### 2.2 Immunochemistry

The surgically resected specimens are fixed with 4% neutral formaldehyde fixative, and then routinely dehydrated, embedded in paraffin, and sectioned. These were sectioned (1 μm) for immunostaining using a Ventana‐automated immunohistochemistry system (BenchMark ULTRA Roche Diagnostics, USA). Sections were stained for ER, PR, Ki‐67, and HER‐2. The reagents for HER‐2 were procured from Roche Diagnostics. The antibodies of ER, PR and Ki‐67 were purchased from Fuzhou Maixin Biotechnology Development Co., Ltd. All primary antibodies are of rabbit origin. Regarding the ER and PR, more than 1% of cells with nuclear staining was considered positive. Record the percentage of HER‐2 membrane staining positive tumor cells. The HER‐2 grade was interpreted according to the intensity of staining and the percentage of stained cells. No staining or ≤10% of infiltrating cancer cells exhibiting incomplete, weak membrane staining are negative (0); The staining was interpreted as mild (1+) when >10% of infiltrating cancer cells present with incomplete and weak cell membrane staining; There are two cases when the staining was graded as moderate (2+). The first is that >10% of infiltrating cancer cells show weak to moderate intensity of intact cell membrane staining; The second is that ≤10% of infiltrating cancer cells show strong and complete cell membrane staining. The staining was graded as strong (3+) when >10% of infiltrating cancer cells show strong, complete and uniform cell membrane staining. Cases with 3+ scores were considered positive, HER‐2 cases scored + or 0 were assessed to be negative. Cases scored 2+ need to be performed fluorescence in situ hybridization (FISH).

### 2.3 Lymph node status

The specimens were fixed with 4% neutral formaldehyde fixative, routinely dehydrated, embedded in paraffin, sectioned and then stained with HE, and observed under light microscope. Cancer cells visible in the pathological section of the lymph nodes are considered to be positive for lymph node tumor metastasis. Also, lymph nodes containing isolated tumor cells are also judged as positive. On the contrary, if there is no tumor cell in the pathological section of the lymph node, it is considered that the lymph node has no metastasis. Using lymph node status positive or negative to mention that.

### 2.4 Molecular subtypes

Molecular subtypes were grouped as follows: luminal A subtype (ER+ or PR+, HER‐2−, and Ki‐67 index [percentage of Ki‐67 tumor cells] <15%), luminal B subtype (ER+ or PR+, HER‐2±, and Ki‐67 index >15%); HER‐2 subtype (ER− and PR−, HER‐2 +), basal‐like subtype (ER−, PR−, and HER‐2−).^22^

### 2.5 Statistical analysis

Statistical analysis was performed by the IBM SPSS Statistics for Windows, Version 23.0 (Armonk, NY: IBM Corp.). Calculate the frequency and percentage of all nominal variables. Summary statistics are provided in Table 1. Categorical variables for molecular subtypes were compared using the chi‐square test. A *p*‐value of <0.05 was considered statistically significant.

**TABLE 1:** Clinicopathological characteristics of molecular subtypes of Paget's disease of the breast (PDB).

Characteristic	Luminal (*n* = 9)	HER2‐enriched (*n* = 15)	Basal‐like (*n* = 2)	Total (*n* = 26)	*p*‐value
Age (years)					
≤50	2	6	0	7	0.553
>50	7	9	2	19	
Breast carcinoma					
DCIS	2	9	0	11	0.087
IDC	7	6	2	15	
Lymph node status					
Positive	6	2	1	9	0.020
Negative	3	13	1	17	

*Note:* For patients with bilateral breast carcinoma, the frequency of axillary lymph node metastasis of PDB in different molecular subtypes was significantly different only information on the side with PDB is listed. For patients with recurrence, only information on the first onset was included.

## 3 RESULTS

Forty‐two patients (all women) with PDB were included in our study. The average patient age at the time of medical treatment was 57 years (range, 39–80 years). Initially, 46 pathology reports were identified, because there were two patients with bilateral breast cancer and 2 with recurrent lesions. Because of technical problems (e.g., tissue blocks were unavailable, or an absence of PCs on the immunohistochemistry sections), 6 patients were excluded. Therefore, 36 patients were included in final analysis. The tumors in 11 patients (31.4%) were located in the right breast, and the tumors in 22 patients (61.1%) were located in the left breast. Two patients (5.7%) had bilateral breast cancer, and in another 1 (2.9%) case the location was undetermined. Of the two relapsed patients, one patient with DCIS relapsed as ipsilateral Invasive ductal carcinoma (IDC) after 6 years; in another patient, the disease relapsed in the form of ipsilateral DCIS after 1 year.

Four of 36 patients had tumors confined to the nipple, and no breast mass was found. 32 patients had tumors with a palpable breast mass. 15 (46.9%) cases of breast lesions were associated IDC, including two cases of bilateral breast cancer and 1 associated with a recurrent mass. There were 17 (53.1%) cases of breast lesions associated with DCIS. There are two special cases are associated with DCIS, pathological primary and recurrent tumors in 1 relapsed patient, and a primary tumor in another relapsed patient. 5 of the 15 IDC s (33.3%) were high‐grade.

Of the 32 patients with a palpable breast mass, we obtained 36 pathological specimens. Cause there are two patients with bilateral breast cancer and two patients are recurring tumors.15 of 36 tumors (41.7%) expressed ER, and 8 (22.2%) expressed PR. 28 patients (77.8%) had a Ki‐67 index greater than or equal to 15%. In 17 of 36 tumors (47.2%), the PCs were immunohistochemically strongly positive for HER‐2, while 12 (19.4%) exhibited negative HER‐2 expression. Another 7 of 36 showed not typical HER‐2 expression and were deleted in subsequent studies. One case of bilateral breast cancer, 1 tumor on the left breast showed high‐level HER‐2 amplification, and 1 tumor on the right breast showed weak HER‐2 expression. The other one case of bilateral breast cancer, both tumors showed negative HER‐2 expression. Two patients with unilateral breast cancer recurrence were included in the study. One of the two patients showed positive HER‐2 expression at first onset and negative HER‐2 expression at the time of recurrence.

**FIGURE 1:**
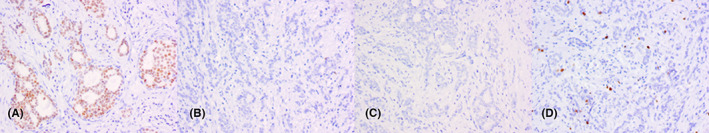
Luminal A‐type PDB positive for ER and PR with weak expression of HER‐2. (A) ER, (B) PR, (C) HER‐2, (D) Ki‐67. Magnification 200 X.

**FIGURE 2:**
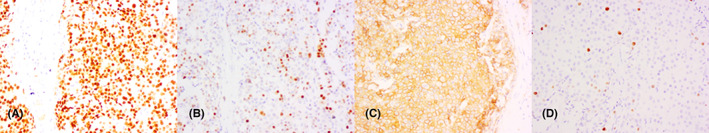
Immunohistochemistry results for luminal B‐type PDB showing the expression of ER and HER‐2 and high proliferation. (A) ER, (B) PR, (C) HER‐2, (D) Ki‐67. Magnification 200 X.

**FIGURE 3:**
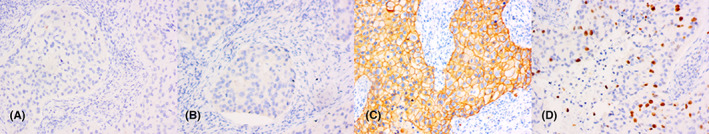
Examples of HER‐2‐type PDB negative for ER and PR and showing expression of HER‐2. (A) ER, (B) PR, (C) HER‐2, (D) Ki‐67. Magnification 200 X.

**FIGURE 4:**
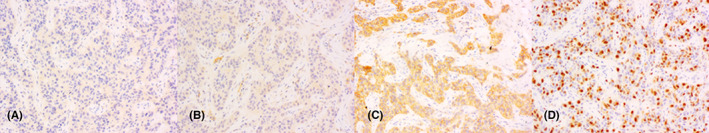
Triple‐negative subtype PDB negative for ER and PR with weak expression of HER‐2. (A) ER, (B) PR, (C) HER‐2, (D) Ki‐67. Magnification 200 X.

For 30 of 36 pathological specimens (83.3%), we obtained a complete molecular subtype through its immunohistochemical reports. Cause the other 6 pathological specimens have missing immunohistochemistry items. Out of the 30 PDB samples, 4 (13.3%) were classified as luminal A (Figure 1), 8 (26.7%) were classified as luminal B (Figure 2), 15 (50.0%) as HER‐2‐enriched (Figure 3), and 3 (10%) as basal‐like sub‐type (Figure 4).

Clinicopathological characteristics of the different molecular subtypes of PDB are listed in Table 1. Information is listed for only one side of the PDB in patients with bilateral breast cancer. For patients with recurrence, only information on the first onset was included. The table compares the molecular subtypes of 26 specimens. Because we try to avoid the discrepancies affecting the final research, which are the different pathological types of bilateral breast carcinoma and the different pathological types of recurrent breast carcinoma from the original lesion. In 9 patients (34.6%), lymph node status was positive; two of these samples were also HER‐2‐enriched. In 17 patients (65.4%), lymph node status was negative. Thirteen out of 17 samples were HER‐2‐enriched. The frequency of axillary lymph node metastasis of PDB in different molecular subtypes was significantly different (*p* = 0.020 < 0.05). However, There was no significant difference in the age distribution (≤50 years or >50 years) of PDB patients with different molecular subtypes. (*p* > 0.05).

## 4 DISCUSSION

In our study lymph node status is relevant to the molecular types of breast cancer, 9 (34.6%) PDB patients had positive lymph nodes, and 17 (65.4%) were negative. The luminal type included more cases with positive lymph nodes. On the contrary, 15 HER‐2 enriched cases usually have 2 cases with positive lymph nodes. This conclusion is different from the study of Arain.^23^ The frequency of axillary lymph node metastasis of PDB in different molecular subtypes was not significantly different in their study. This difference may be due to bias caused by insufficient sample size. This problem is unavoidable, because PDB patients are rare. By retrieving 10‐year records, we only collected 42 PDB patients from more than 2000 breast cancer patients. Due to the imperfections of previous immunohistochemistry projects, we only collected the complete molecular subtypes of 26 specimens.

Consistent with a recent study,^12^ HER‐2 amplification was more prevalent in our cohort of PDB patients than it was in breast carcinoma. Triple‐negative PDB cases exist but are very rare. We found two cases (7.7%) of triple negative PDB phenotype in our research. In another large PDB study that identified PDB molecular subtypes, no triple‐negative cases were observed, but They found that 86% of the cases were the HER‐2 subtype.^12^ In our study, the HER‐2 subtype accounted for 57.7% of the patients, and both cases of triple‐negative PDB were accompanied by invasive carcinoma.

Diagnosis of PDB should prompt a thorough examination of the breast, since in many cases PDB is accompanied by DCIS and/or ICB. The breast should be thoroughly checked for other lesions to avoid incomplete excision of skin tumor in situ. In this study, 17 (53.1%) PDB specimens had DCIS and 15 (46.9%) had IDC. In another study of 55 patients, Onoe identified 23 DCIS patients (42%) and 32 (58%) IDC patients.^24^ Song's team found breast cancer in 57 of 66 patients with PDB.12 (21%) out of 57 breast cancers were DCIS, while 45 (79%) were IDC.^25^

In our study, two patients with bilateral breast cancer had different molecular types on both sides, and only one side had Paget's disease. Meanwhile, there are two patients with recurrence. The molecular classification of primary tumor and recurrent tumor in one case was also different. Several mechanisms have been proposed for changes in biomarker expression between primary and recurrent BC: intratumoral heterogeneity^26^, ^27^ and selection pressure from prior therapy.^28^, ^29^ Also, changes in individual genes may occur.^30^, ^31^ The primary tumor is PDB, which recurred after surgical removal of NAC. Therefore, we think that the margins of surgical resection of tumors may require finer standards to avoid tumor recurrence for PDB patients.

93% of PDB‐related cancers in Lester's group were high nuclear grade,^32^ whereas all IDC cases in Kothari's study were high grade.^33^ In their studies, PDB was highly correlated with high‐grade IDC, consistent with its aggressive clinical behavior.^32^, ^33^ In our study, 5 of the 15 ICDs (33.3%) were high‐grade. Our research results do not seem to be similar to the results of Kothari's research.^33^ We are not sure whether this difference is due to genetic differences in the populations sampled or bias caused by the small sample size.

Overall, breast cancer is HER‐2 positive in 13%–30% of cases^34^ and HER‐2 positivity is higher in PDB and related breast cancers, ranging from 60% to 80%.^34^, ^35^, ^36^, ^37^, ^38^ In our study, 15 (57.7%) PDB samples were HER‐2 positive (score 3+).

**FIGURE 5:**
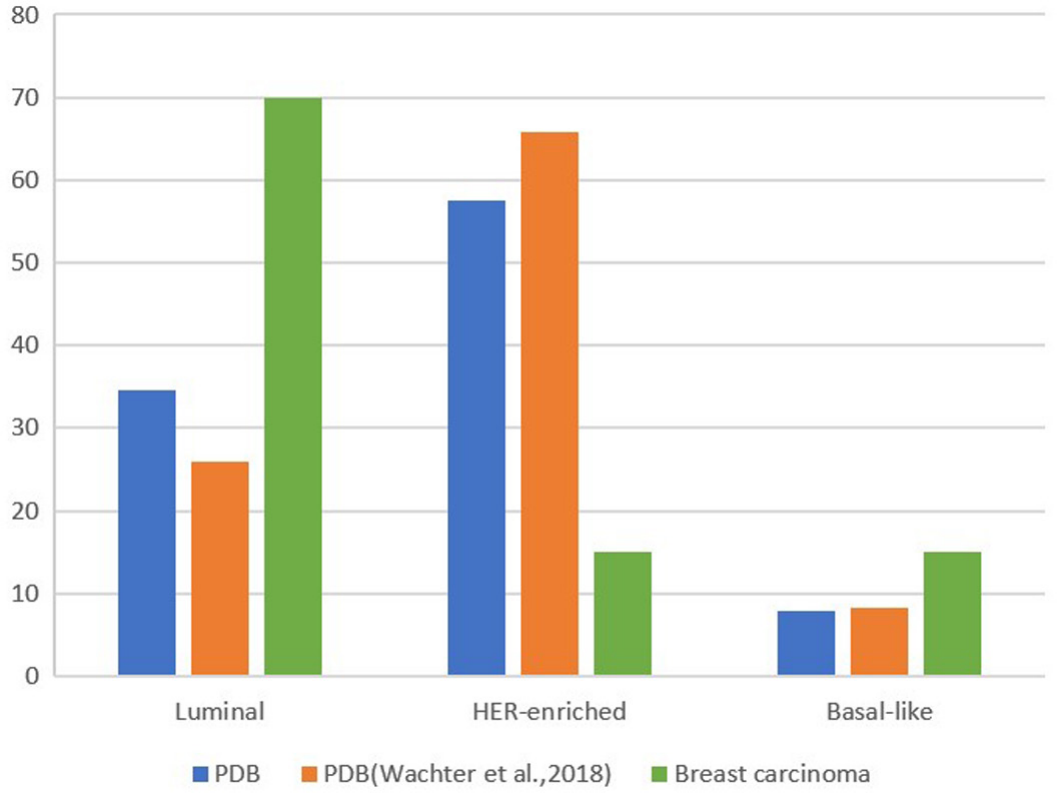
Distribution of the molecular subtypes of PDB in this study and of PDB^36^ and breast carcinoma^39^ according to the literature.

The distribution of the molecular subtypes in PDB and breast cancer in general from this and other studies are depicted (Figure 5).Comparison of the studies reveals significant differences in the molecular subtypes between PDB and breast cancer in general. The luminal subtype is the most common molecular subtype of common breast cancer. In contrast, the HER‐2‐positive subtype is more common in two PDB studies. HER‐2‐positive subtype is more aggressive and has a worse prognosis. A high proportion of HER‐2‐positive subtype is associated with worse prognosis in PDB.^33^, ^35^ A limitation of our study was the small number of cases due to the rarity of PDB. Due to the rarity of PDB, our study was limited by the small number of cases. Fortunately, this sample size was able to assess statistical significance, and our results clearly show a higher frequency of HER‐2‐positive subtype.

## 5 CONCLUSION

Our study showed that the HER‐2‐positive subtype was the most common molecular subtype of PDB, which was related with breast cancer and was more likely to be related with high‐grade IDC. Molecular subtypes vary between PDB and general breast carcinomas. In contrast to the most common luminal subtype in nonspecific breast cancer, the HER‐2‐positive subtype is the most common subtype in PDB and related breast cancer. The frequency of axillary lymph node metastasis of PDB in different molecular subtypes was significantly different (*p* < 0.05).Recognizing the HER‐2 as the dominant subtype of PDB and related breast cancer should prove useful as a diagnostic tool and may assist in development of therapeutic protocols and cures for this disease will be achieved.

## AUTHOR CONTRIBUTIONS

**Yujiao Cai:** Conceptualization (equal); data curation (equal); formal analysis (equal); methodology (equal); project administration (equal); supervision (equal); validation (equal); visualization (equal); writing – original draft (equal); writing – review and editing (equal). **Zhongquan Cheng:** Data curation (equal); formal analysis (equal); software (equal); supervision (equal); writing – review and editing (equal). **Jiarui Nangong:** Data curation (equal); formal analysis (equal); writing – review and editing (equal). **Xiaodan Zheng:** Conceptualization (equal); data curation (equal); project administration (equal); resources (equal); supervision (equal). **Zhu Yuan:** Conceptualization (equal); resources (equal); software (equal); supervision (equal); writing – review and editing (equal).

## FUNDING INFORMATION

None.

## CONFLICT OF INTEREST STATEMENT

We have no conflict of interest with the products or companies described in this article.

## ETHICAL APPROVAL

This study was reviewed and approved by Ethics Committee of Beijing Friendship Hospital, Capital Medical University. All participants signed informed consent. Our research follows the Declaration of Helsinki.

## CONSENT

All authors agree to publish.

## Data Availability

The data used in this study can be obtained from the corresponding author under reasonable request.
